# Development of a Green Microwell Spectrofluorimetric Assay with High Analytical Throughput for the Determination of Selective Serotonin Reuptake Inhibitors in Pharmaceutical Dosage Forms and Plasma

**DOI:** 10.3390/molecules28135221

**Published:** 2023-07-05

**Authors:** Nourah Z. Alzoman, Ibrahim A. Darwish

**Affiliations:** Department of Pharmaceutical Chemistry, College of Pharmacy, King Saud University, P.O. Box 2457, Riyadh 11451, Saudi Arabia; nalzoman@ksu.edu.sa

**Keywords:** SSRIs, spectrofluorimetry, microwell assay, green and high-throughput analysis, pharmaceutical and biomedical applications

## Abstract

In this study, a new green microwell spectrofluorimetric assay (MW-SFA) with high throughput was developed and validated, for the first time, for the determination of three selective serotonin reuptake inhibitors (SSRIs) in pharmaceutical dosage forms and plasma. These SSRIs were fluoxetine (FLX), fluvoxamine (FXM), and paroxetine (PXT), which are commonly prescribed drugs for depression treatment. The MW-SFA is based on the condensation reaction of SSRIs with 4-chloro-7-nitrobenzo-2-oxa-1,3-diazole (NBD-Cl) in alkaline media to form highly fluorescent derivatives. The MW-SFA procedures were conducted in 96-microwell white opaque assay plates with a flat bottom and the fluorescence signals were measured using a microplate reader at their maximum excitation and emission wavelengths. The calibration curves were generated with good correlation coefficients (0.9992–0.9995) between the relative fluorescence intensity (RFI) and the SSRI concentrations in the range of 35–800 ng/mL. The limits of detection were in the range of 11–25 ng/mL, and the precision and accuracy were satisfactory. The proposed MW-SFA was successfully applied to the analysis of the SSRIs in their pharmaceutical dosage forms. The statistical analysis for the comparison between the MW-SFA assay results and those of pharmacopeial assays showed no significant differences between the assays in terms of their accuracy and precision. The application of the proposed MW-SFA was extended to successfully analyze SSRIs in plasma samples. The greenness of the assay was confirmed using three different metric tools. The assay was characterized with high throughput properties, enabling the sensitive simultaneous analysis of many samples in a short time. This assay is valuable for rapid routine applications in pharmaceutical quality control units and clinical laboratories for the determination of SSRIs.

## 1. Introduction

Depression is a mental health condition that is widespread and affects approximately 5% of adults globally. It is caused by alterations in certain central neurotransmitter levels and/or their biochemical functions. These changes in neurotransmitter balance, such as norepinephrine, serotonin, or both, are due to impaired synthesis of neurotransmitters, increased metabolism or breakdown, and/or increased pump uptake [1]. To manage depression, the most commonly used approach is the use of antidepressant drugs. These drugs work by reducing the reuptake of neurotransmitters and the production of cyclic adenosine monophosphate, which is involved in depression pathogenesis. There are four main classes of antidepressant drugs: tricyclic antidepressants, monoamine oxidase inhibitors, selective serotonin reuptake inhibitors (SSRIs), and other miscellaneous antidepressants [2].

SSRIs are commonly recommended as the first-line therapy for depression and other psychiatric conditions due to their safety, effectiveness, and tolerability. These drugs work by selectively inhibiting the reuptake of serotonin by presynaptic receptors, resulting in increased serotonin levels in the synapse and the stimulation of the CNS. The FDA has granted approvals for nine SSRIs for the treatment of depression, including fluoxetine, fluvoxamine, paroxetine, sertraline, citalopram, escitalopram, vilazodone, dapoxetine, and vortioxetine. These drugs have diverse chemical structures that affect their physicochemical and pharmacokinetic properties [3]. Although SSRIs have similar antidepressant potency to tricyclic antidepressants, they are preferred by patients due to their superior safety profile, lack of sedative effects, and long half-life, which allows for once-daily or once-weekly administration. These advantages have contributed to the widespread and successful use of SSRIs as antidepressant medications [4].

The exact active drug content and content uniformity of pharmaceutical dosage forms are crucial for ensuring the effectiveness and safety of SSRIs in treating depression. Therefore, it is essential to conduct a proper assay for quality control purposes in order to meet these requirements. In addition, the determination of plasma SSRI levels is necessary because these levels control the serotonin level in the patient’s body, change serotonin reuptake transporter binding and shelter-seeking behavior, and accordingly control the efficiency of SSRIs as antidepressant drugs [5]. Various assays utilizing different instrumentations have been proposed for the analysis of dosage forms of SSRIs and reviewed in several articles [6,7]. Among these assays, spectrofluorimetric assays are commonly used [8,9,10,11,12,13,14,15,16,17,18,19]. Spectrofluorimetric assays were reported for paroxetine [8], sertraline [11], fluvoxamine [12], and fluoxetine [17,20]. Spectrofluorimetric assays are preferred due to their high sensitivity, simplicity, convenience, interfacing to automated spectrofluorimetric analyzers, and/or applicability to the analysis of dosage forms and biological samples. However, the existing spectrofluorimetric assays for SSRIs use conventional analytical practices such as volumetric flasks/cuvettes and manual analysis, limiting their throughput and, thus, not meeting the needs of quality control laboratories processing large batches of pharmaceutical formulations [21,22,23]. In addition, the reported spectrofluorimetric assays for SSRIs were developed individually: one assay for one SSRI. However, the availability of one assay that is valid for all SSRIs would be very convenient. Moreover, the assays that have been reported require significant quantities of organic solvents, which can be expensive, ecologically damaging, and may pose a risk to the health of analysts [20,24,25]. Consequently, these assays do not meet the expectations of pharmaceutical companies for high-throughput assays [21,22,23] and fail to adhere to the principles of the green analytical chemistry (GAC) approach [26].

**Figure 1:**
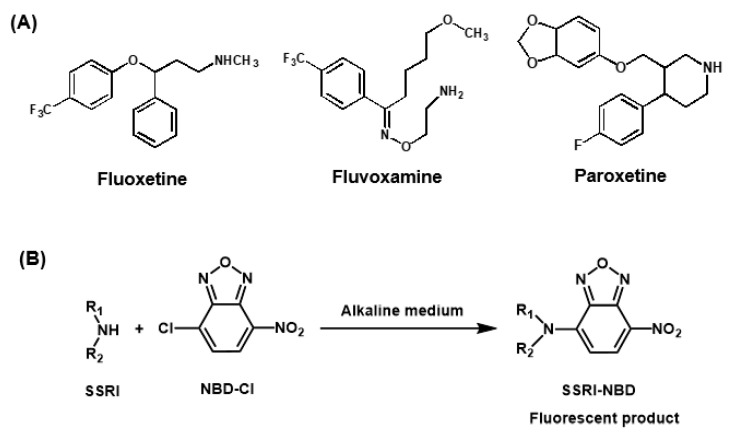
Panel (**A**): chemical structures of SSRIs and their abbreviations. Panel (**B**): chemical reaction of SSRIs with NBD-Cl reagent.

This study details the development of a novel microwell spectrofluorimetric assay (MW-SFA) for the determination of SSRIs in pharmaceutical dosage forms and plasma samples. The assay was established using three representative examples of SSRIs: fluoxetine (FLX), fluvoxamine (FXM), and paroxetine (PXT). These drugs were chosen because they are widely prescribed for depression treatment [27] and are listed in the British Pharmacopoeia [28] and United States Pharmacopeia [29]. In addition, these SSRIs represent the different functional groups (primary and secondary amino groups) encountered in most SSRIs. The chemical structures of FLX, FXM, and PXT are shown in Figure 1. The MW-SFA involved the condensation reaction of SSRIs, via their primary or secondary amino groups, with 4-chloro-7-nitrobenzo-2-oxa-1,3-diazole (NBD-Cl) in alkaline media, resulting in the formation of fluorescent reaction products. The reaction was conducted in 96-well white opaque assay plates, and the fluorescence signals of the reaction products were measured using a microplate reader in a fluorescence mode. The proposed MW-SFA meets the principles of the GAC approach and fulfils the requirements of high-throughput analysis for pharmaceutical and clinical analysis.

## 2. Results and Discussion

### 2.1. Strategy of Assay Development, Reaction Involved, and Fluorescence Spectra

**Figure 2:**
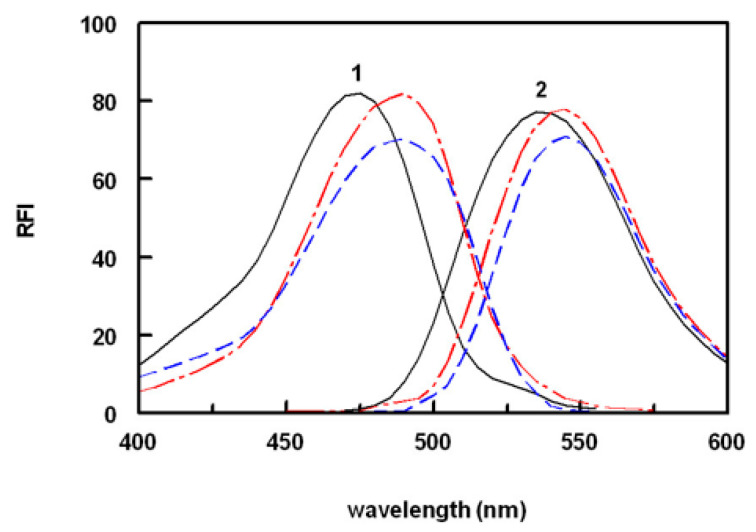
Excitation (1) and emission (2) spectra of the reaction product of NBD-Cl with FXM (black), FLX (red), and PXT (blue). RFI is the relative fluorescence intensity. The concentrations of SSRIs were 400, 300, and 300 ng/mL for FXM, PXT, and FLX, respectively.

SSRIs do not exhibit native fluorescence signals that are adequate for their direct sensitive spectrofluorimetric analysis. To increase their detectability for spectrofluorimetric determination, it was necessary to derivatize them with derivatizing reagents. Different reagents have been proposed for their derivatization, mostly targeting their primary or secondary amino groups for derivatization [30]. Among these reagents, NBD-Cl was chosen as the derivatizing reagent in the present study because it can form highly fluorescent derivatives with both primary (FXM) and secondary (FLX and PXT) amines under relatively mild reaction conditions. In the present study, a fresh solution of NBD-Cl was prepared daily due to the presence of labile chloride in its chemical structure. The investigated SSRIs were reacted with NBD-Cl, and found to form yellow-colored fluorescent derivatives that exhibited maximum fluorescence intensity (λ_em_) at 535 nm for FXM and 545 nm for FLX and PXT upon excitation at a maximum wavelength (λ_ex_) of 470 nm for FXM and 490 nm for FLX and PXT. The reaction pathway is given in Figure 1B, and the excitation and emission spectra for the reaction products of NBD-Cl with FXM, FLX, and PXT are given in Figure 2. According to these findings, spectrofluorimetric measurements for the optimization of reaction variables in microwell assay plates were performed at these wavelengths.

### 2.2. Optimization of Reaction Variables

#### 2.2.1. Effect of NBD-Cl Concentration

**Figure 3:**
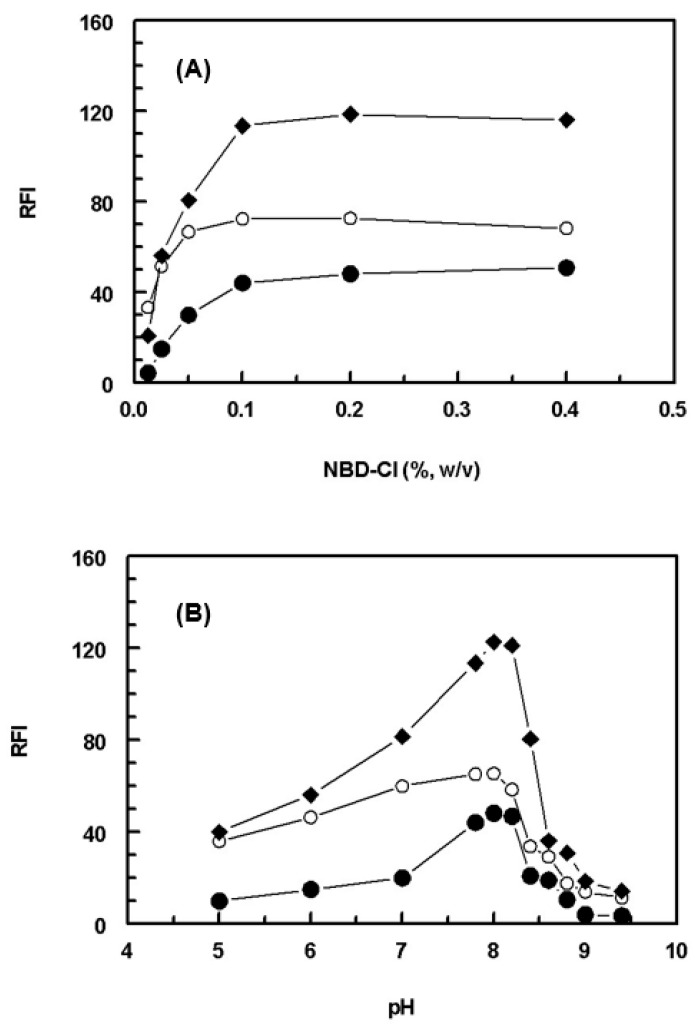
Effect of NBD-Cl concentrations (**A**) and pH of the reaction medium (**B**) on the reaction of NBD-Cl with FXM (●), PXT (○), and FLX (◆). The concentrations of SSRIs were 400, 600, and 200 ng/mL for FXM, PXT, and FLX, respectively. RFI is the relative fluorescence intensity.

Studying the effect of NBD-Cl concentration on its reaction with SSRIs revealed that the reactions were dependent on NBD-Cl concentration as the RFI values of the reaction solutions increased as the NBD-Cl concentration increased (Figure 3A). The highest RFI readings were attained at a NBD-Cl concentration of 0.1% (*w*/*v*), beyond which no further increase in RFI was observed upon further increase in NBD-Cl concentrations up to 0.4%. For obtaining higher precision in the readings, further experiments were carried out using 0.2% for the three SSRIs.

#### 2.2.2. Effect of pH

In order to initiate and accelerate the nucleophilic condensation reactions between NBD-Cl and SSRIs, nucleophiles should be generated by carrying out the reactions in alkaline media. The effect of the pH of the reactions was studied by carrying out the reactions in Clark and Lubs buffer solution of different pH values in the range of 5–9.5. The results revealed that the reactions were dependent on the pH values (Figure 3B). The RFI values increased as the pH increased and maximum RFI values were attained when the pH was 8 ± 0.2. This parallel relation between the RFI and pH was attributed to the transformation of the amino groups of SSRIs from their acid salt forms (in acidic pH values) to their corresponding free amino group forms (in alkaline pH). The existence of SSRIs in their free amine forms facilitated their nucleophilic condensation reactions with NBD-Cl. At pH higher than 8.2, a dramatic decrease in the RFI readings occurred. This was attributed to the high concentration of hydroxide ions, which holds back the condensation reaction between SSRIs and NBD-Cl. According to these findings, the subsequent experiments were carried out at pH 8 ± 0.2.

#### 2.2.3. Effect of Temperature and Time

To establish the optimum temperature and time required for the achievement of the reactions, the reactions were allowed to proceed for varying time periods at different elevated temperatures (25–50 °C). These temperatures were maintained by the built-in temperature control system of the microplate reader. It was able to incubate the assay plate for the desired time at the selected temperature and monitor the RFI readings at different time intervals. It was found that the reactions were very slow at room temperature (25 ± 2 °C) and did not reach completion in a reasonable time; they required more than 1 h. At higher temperatures, the reactions were accelerated, and they reached completion in 20 min at 50 °C. Therefore, further experiments were conducted by incubating the assay plates for 20 min in the microplate reader whose temperature was set at 50 °C. Temperatures higher than 50 °C were not tested because the highest temperature that can be achieved by the built-in temperature control of the reader is 50 °C.

#### 2.2.4. Effect of Solvent

**Figure 4:**
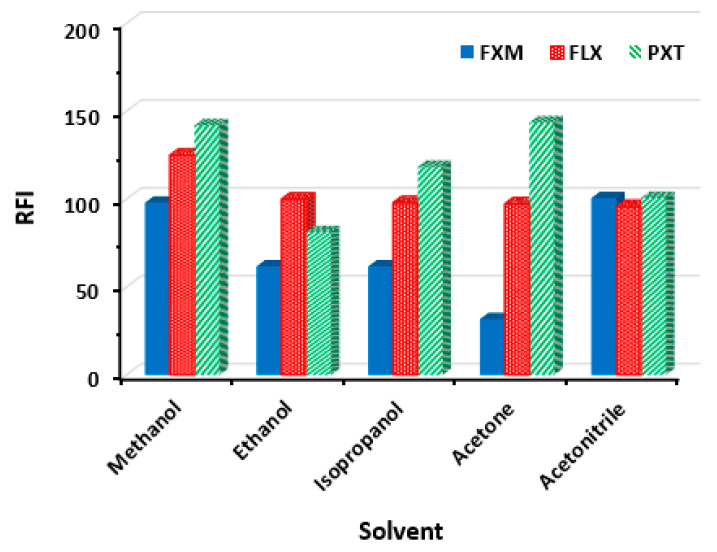
Effect of solvent on the reaction of NBD-Cl (0.2%, *w*/*v*) with FXM, FLX, and PXT. The concentrations of SSRIs were 400, 600, and 200 ng/mL for FXM, PXT, and FLX, respectively. RFI is the relative fluorescence intensity.

The influence of solvent was investigated by executing the reaction in different solvents. These solvents were water, methanol, ethanol, isopropanol, acetone, and acetonitrile. When using water, a colloidal appearance was observed in the reaction wells, indicating the incomplete solubility of SSRI-NBD fluorescent derivatives in water. Upon using the other solvents, different RFI values were attained (Figure 4). For FXM, comparably high RFI values were obtained when methanol and acetonitrile were used. However, the GAC principle [26] recommends methanol as a green solvent rather than acetonitrile. Therefore, methanol was selected for FXM involving experiments. For FLX, the optimum solvent was methanol (Figure 4). For PXT, the highest comparable RFI values were obtained when methanol and acetone were used. For green solvent consideration [26], methanol was selected for its subsequent experiments. In conclusion, methanol was used as a solvent for the subsequent experiments involving all SSRIs.

#### 2.2.5. Effect of HCl Concentration

**Figure 5:**
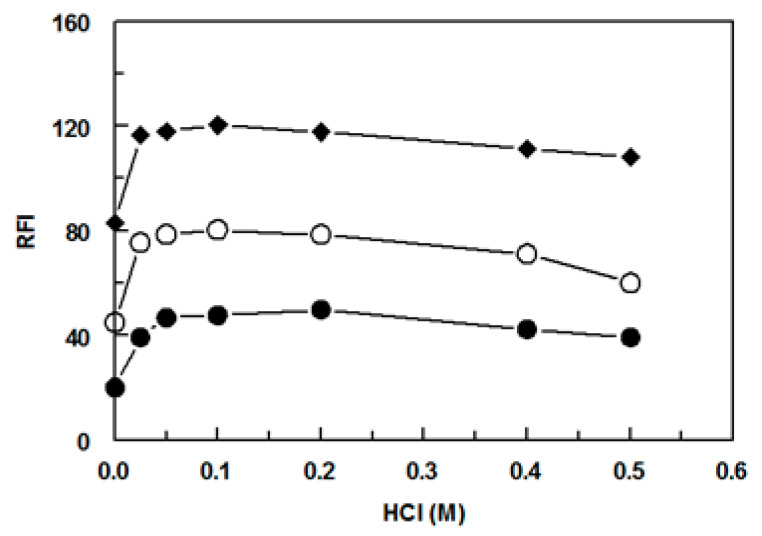
Effect of HCl concentration on the fluorescence intensity of the reaction product of NBD-Cl with FXM (●), PXT (○), and FLX (◆). The concentrations of SSRIs were 400, 600, and 200 ng/mL for FXM, PXT, and FLX, respectively. RFI is the relative fluorescence intensity.

Under the aforementioned established reaction conditions, high RFI values were obtained in the blank wells. This was due to the documented hydrolysis of NBD-Cl to its hydroxy derivative (NBD-OH) [31]. To minimize the fluorescence signals of the blank wells and ultimately enhance the net signals and assay sensitivity, the fluorescence of NBD-OH should be quenched. It was reported that this quenching could be accomplished by decreasing the pH of the reaction medium to one pH unit [32]. The concentration of HCl required for acidification was studied in the range of 0.025 to 0.5 (M), and it was found that the optimum net RFI values was attained when 50 μL of HCl (0.1 M) was added to each well of the assay plate (Figure 5). HCl was added after the completion of the reaction of the SSRIs with NBD-Cl to quench the fluorescence of NBD-OH. This concentration was used for the subsequent experiments. The RFI of the blank wells were similar to those obtained with 0.025 M (Figure 5).

#### 2.2.6. Stability of the Fluorophore

The influence of time on the stability of the fluorescent SSRI-NBD reaction products was investigated by monitoring the RFI of the reaction solutions over a time period. It was discovered that the RFI values persist unchanged for at least 1 h, beyond the completion of the reactions. This allowed the manipulation and processing of many batches of samples, and their comfortable measurements with convenience. This increased the convenience of the assay as well as made it applicable for a large number of samples.

A summary of the establishment of optimum conditions for the reaction of NBD-Cl with the investigated SSRIs is given in Table 1.

### 2.3. Validation of MW-SFA

#### 2.3.1. Calibration and Sensitivity

To construct the calibration curves for the determination of SSRIs, their reactions with NBD-Cl were performed under their optimum conditions (Table 1), and the measured RFI values were plotted as a function of the corresponding concentrations of SSRI. It was found the RFI values are in a linear relationship with the SSRI concentration in the concentration ranges of 65–800, 35–500, and 80–800 ng/mL for FXM, FLX, and PXT, respectively. The regression equation for the results was RFI = a + b C, where RFI is the relative fluorescence intensity, a is the intercept of the calibration line, b is the slope, and C is the concentration of SSRI in ng/mL. For the three SSRIs, linear relationships with small intercepts and good correlation coefficients were obtained (Table 2). The limits of detection (LOD) and limit of quantitation (LOQ) were determined according to the International Council for Harmonisation (ICH) guidelines for the validation of analytical procedures [33]. The LOD values were 21, 11, and 25 ng/mL for FXM, FLX, and PXT, respectively. The LOQ values were 64, 33, and 77 ng/mL for FXM, FLX, and PXT, respectively.

The analytical performance of the proposed MW-SFA is summarized in Table 2.

#### 2.3.2. Accuracy and Repeatability

The accuracy of MW-SFA was assessed by the recovery studies for added SSRI concentrations. The recovery values were 97.4–102.4 ± 1.06–2.55% (Table 3), indicating the accuracy of the proposed MW-SFA.

The repeatability of the proposed MW-SFA was evaluated by the replicate analysis of five separate solutions of the working standards of SSRIs. The assay gave satisfactory results; RSD did not exceed 3.37%, indicating the good repeatability of the results obtained by the proposed MW-SFA (Table 4). This precision level is adequate for the precision and routine analysis of the investigated drugs. This high level of result repeatability was attributed to the accurate dispensing of the SSRI samples and other reagents using multi-channel pipettes.

#### 2.3.3. Selectivity

The selectivity of the proposed MW-SFA was tested by applying its refined established procedure to the analysis of the investigated SSRIs in the presence of the common pharmaceutical excipients that are frequently used during SSRI formulation. The samples were prepared by mixing known quantities of SSRI (50 mg of FXM and 20 mg of FLX and PXT) with different quantities of the excipients: starch, glucose, lactose, talc, and magnesium stearate. The recovery values were 97.0–103.0 ± 1.25–2.81%. This result revealed the absence of interference from any of the common excipients with the determination of SSRIs by the proposed MW-SFA. This high selectivity was attributed to the selective reaction of the amino groups of SSRIs with NBD-Cl forming fluorescent derivatives leaving the non-fluorescent excipients.

#### 2.3.4. Robustness and Ruggedness

The robustness was evaluated by conducting the assay procedure under small deviations from the optimum procedures. In this set of experiments, one factor was changed while the others were kept constant, and the recovery (%) was calculated. It was found that the variation in the NBD-Cl concentrations (0.15–0.25%, *w*/*v*), temperature (optimum ± 2 °C), and time (optimum ± 5 min) did not significantly affect the assay performance; the recovery values were 97.6–103.1, and the RSD values did not exceed 3%. The most critical parameter affecting the results was the pH; it should be adjusted to 8 ± 0.2.

The ruggedness of the assay was also checked by applying the assay to analyze the SSRIs using the same procedures, but on two instruments at two laboratories and at three days. The results obtained from instrument to instrument and day to day were reproducible, as the relative standard deviations (RSD) did not exceed 3.34% (Table 5).

### 2.4. Applications of MW-SFA to the Analysis of Dosage Forms and Plasma Samples

The MW-SFA proposed in this study was utilized for analyzing the investigated SSRIs in their dosage forms. The label claim percentages were within the range of 99.2–100.2 ± 1.32–1.83% (Table 6). The same samples were also analyzed using the official assays [28,29] employing chromatographic techniques, and the results obtained from both methods were compared through statistical analysis to determine their accuracy and precision using a *t*- and F-test, respectively. The data showed that there were no significant differences between the practically found and theoretical values of the *t*-test and F-test at a 95% confidence level, indicating similar accuracy and precision in determining the investigated SSRIs using both assays. It is wise to mention that the proposed MW-SFA has simpler procedures and higher throughputs than the official chromatographic assays [28,29]. In addition, the proposed assay has much higher sensitivity than the official methods; therefore, the applicability of the proposed assay was enabled.

Due to the adequate sensitivity of the proposed MW-SFA, it was deemed appropriate to investigate its applicability for determining the investigated SSRIs in plasma samples. Because the proposed assay is based on the reaction of the amino groups of SSRIs with NBD-Cl, interferences from biogenic amines in plasma are expected. To eliminate this potential interference, extraction procedures for the SSRIs from plasma samples were conducted before carrying out the reaction between SSRIs and NBD-Cl. The plasma samples were spiked with varying concentrations of SSRIs (80–500 ng/mL) and the extraction was conducted according to the procedures described by Al-Rayes [18]. The results of the analysis of spiked FXM and PXT were satisfactory in terms of accuracy and precision, with recovery values of 97.8–101.4% (±1.54–2.55) and 97.4–102.2% (±1.56–2.97) for FXM and PXT, respectively (Table 7). In the case of FLX, the recovery values were unacceptably high (≥145%). This was attributed to the fact that FLX is metabolized in the liver to an active metabolite, nor-FLX, which is an amine and has a high plasma level. Since both compounds (FLX and nor-FLX) are amines, they can react with the NBD-Cl reagent, resulting in high recovery values. These findings demonstrate the suitability of the proposed MW-SFA for the analysis of plasma containing FXM and PXT; however, it is not suitable for the analysis of samples containing FLX.

### 2.5. Greenness of MW-SFA

Assays utilizing microwell plates and microplate readers typically adhere to the principles of GAC due to their low solvent/reagent consumption and minimal waste production. To accurately evaluate the greenness of the proposed MW-SFA for SSRIs, three different metric tools were utilized, namely, NEMI [26], GAPI [34], and AGREE [35]. The experimental procedures for these tools and their results (pictograms) were outlined in their corresponding published articles.

**Figure 6:**
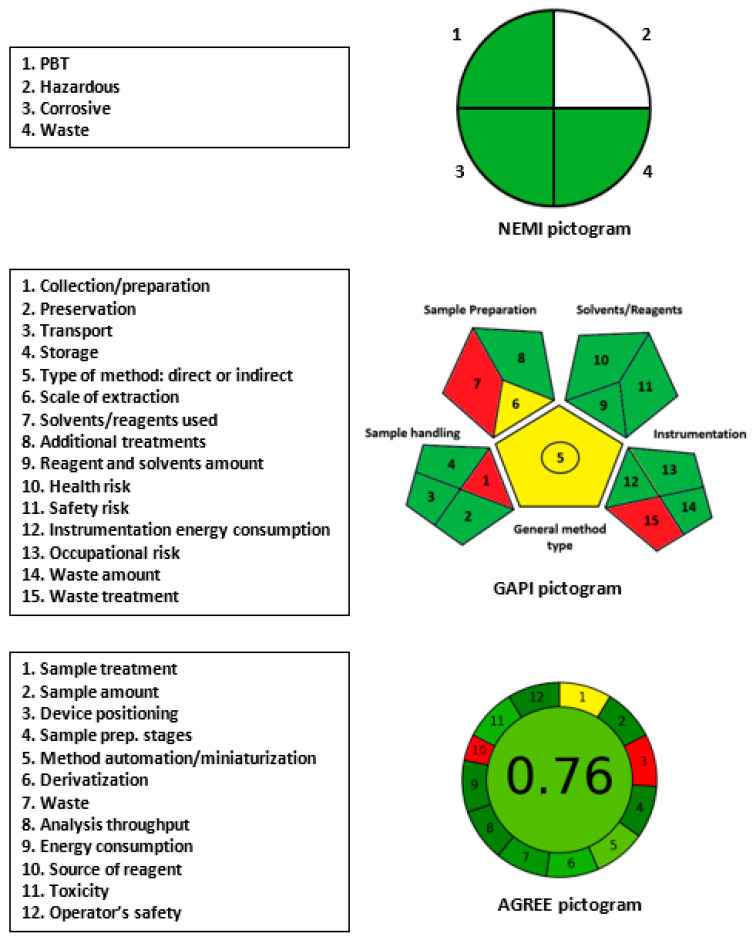
Results of NEMI, GAPI, and AGREE analysis for the evaluation of the greenness of the proposed MW-SFA for SSRIs.

In the NEMI pictogram, the quadrants corresponding to PBT, corrosive, and waste were green, while the hazardous quadrant remained blank (Figure 6). The hazardous quadrant did not turn green because buffer/NaOH and solvents were used in the sample preparation, which are classified as hazardous materials. However, the waste quadrant was green because the proposed method produces small volumes of waste (350 µL per sample).

In the GAPI pictogram (Figure 6), the three parameters corresponding to sample collection/preparation (1), solvents/reagents used (7), and waste treatment (15) were colored red. This outcome was due to the use of non-green solvents/reagents in sample preparation (e.g., buffer/NaOH, extraction solvents, and NBD-Cl), the off-line sample collection/preparation, and the absence of waste treatment.

In the AGREE pictogram (Figure 6), parameters 3 (device positioning) and 10 (source of reagents) were colored red due to the off-line sample treatment and the lack of automated operation of the microplate reader. The other parameters were colored yellow or varying degrees of green. The automatically generated number in the center of the AGREE’s pictogram for overall assessment was 0.76 out of 1, confirming the overall greenness of the proposed assay.

In conclusion, the proposed MW-SFA fulfills the requirements of GAC for routine application in pharmaceutical quality control laboratories and clinical laboratories for the analysis of SSRIs, as confirmed by the overall evaluation of its eco-friendliness and greenness.

### 2.6. High Throughput of MW-SFA

Principally, microwell assays are a powerful tool for high-throughput analysis, enabling the simultaneous processing of large numbers of samples in a cost-effective manner. Here are some key aspects of the proposed MW-SFA that contribute to its high throughput capabilities:

*Resource utilization*: MW-SFA offers high resource utilization efficiencies, as it requires minimal SSRI samples and reagent volumes. This leads to reduced waste and cost savings, making the assay an ideal choice for a large-scale screening tool for SSRIs.

*Processing speed*: MW-SFA has a fast-processing speed, as multiple SSRI samples can be processed in parallel as a batch. An analyst could handle at least six plates as a batch (576 samples) per 30 min (i.e., 1152 samples per working hour). This increases the overall throughput and reduces the time required to complete the analytical process.

*Scalability*: MW-SFA is highly scalable, as it can be easily adapted to accommodate different microwell plates with a higher number of wells such as 384- and 1536-well plates. This makes the assay suitable for varying scales of analysis.

***Cost***: MW-SFA is cost effective, as it requires minimal reagents, and it can be performed using a standard microplate reader.

*Parallel processing*: The assay is designed for parallel processing, enabling the high-throughput analysis of multiple samples simultaneously. This allows for efficient sample analysis and more rapid data acquisition, which is particularly useful in large-scale analysis in pharmaceutical industries of SSRIs.

In summary, the proposed MW-SFA is a powerful tool for the high-throughput analysis of SSRIs, offering efficient resource utilization, fast processing speed, scalability, cost-effectiveness, parallel processing, and high-throughput analysis capabilities. These features make the assay an ideal choice for the analysis of SSRIs in pharmaceutical and clinical laboratories.

## 3. Experimental Section

### 3.1. Instruments

The SpectraMax^®^ M5 microplate reader was manufactured by Molecular Devices, LLC (San Jose, CA, USA). The reader can perform several types of detections including absorbance, fluorescence, fluorescence polarization, time-resolved fluorescence, and chemiluminescence. It can accommodate microplates ranging from standard 6-well to 384-well plates and has a four-zone system for temperature control up to 50 °C, ensuring temperature-sensitive assays can be carried out with excellent stability. The SpectraMax M5 reader is controlled by SoftMax^®^ Pro Enterprise software. All fluorescence spectra were recorded using a FP-6200 fluorometer (JASCO Co., Ltd., Kyoto, Japan), with 1 cm quartz cells used for all measurements. The JB1603-C/FACT Caratbalance digital balance is a product of Mettler-Toledo International Inc., located in Zürich, Switzerland. The pH meter used in the experiments was the Model Jenway 350, manufactured by Bibby Scientific Ltd. (Essex, UK). The Purelab Flex water purification system (ELGA Veolia Ltd., High Wycombe, UK) was utilized to provide the pure water used throughout the study.

### 3.2. Standards, Dosage Forms, and Plasma

The standards of fluoxetine HCl (FLX), fluvoxamine HCl (FXM), and paroxetine HCl (PXT) were obtained from the following manufacturers: Hetero Drugs Ltd. in Hyderabad, India, for FLX, Solvay Pharma in Suresnes, France, for FXM, and SmithKline Beecham Pharmaceuticals in Brentford, England, for PXT. The three drugs are white to almost-white crystalline powders. The following dosage forms of the investigated SSRIs were used: Prozac (Eli Lilly & Co., Ltd., Hampshire, UK), Fluzac (Riyadh Pharma, Riyadh, Saudi Arabia), Salipax (Mepha Ltd., Aesch-Basel, Switzerland), Flutin capsules (Egyptian International Pharmaceutical Industries Co., Cairo, Egypt), and Octozac capsules (October Pharma, S.A.E., Cairo, Egypt); all are labeled to contain 20 mg FLX per capsule. Faverin tablets (Solvay Pharma, Suresnes, France) are labeled to contain 50 mg FXM per tablet. Seroxat tablets (SmithKline Beecham Pharmaceuticals, Brentford, UK) are labeled to contain 20 mg of PXT per tablet. Drug-free plasma was provided by the Blood Bank at King Khalid Hospital (King Saud University, Riyadh, Saudi Arabia). The plasma was stored at −20 °C until analyzed.

### 3.3. Reagents, Buffer Solutions, and Tools

The 4-chloro-7-nitrobenzo-2-oxa-1,3-diazole (NBD-Cl) was purchased from Sigma-Aldrich Chemicals Co. (St. Louis, MO, USA). To prepare a solution of NBD-Cl, 20 mg of the powder was dissolved in 10 mL of methanol to make a 0.2% (*w*/*v*) solution. The solution was freshly prepared and was kept protected from light during use. Clark and Lubs buffer solutions of different pH values were used. To prepare the buffer solution, a mixture was made by combining 50 mL of boric acid and potassium chloride solutions (0.2 M). The pH of the buffer solutions was adjusted by sodium hydroxide (0.2 M). White opaque flat-bottomed microwell plates with 96 wells of 360 µL total volume were acquired from Corning/Costar Inc. (Cambridge, MA, USA). The pipettes were purchased from Sigma-Aldrich Chemicals Co. (St. Louis, MO, USA). The solvents were spectroscopic grade, and were acquired from Fisher Scientific (California, CA, USA).

### 3.4. Preparation of Standard and Sample Solutions

#### 3.4.1. Standard Solutions

An accurate weight (20 mg) of each SSRI was dissolved in 10 mL distilled water to obtain a stock solution of 2 mg/mL. The stock solution was found to be stable for at least two weeks when kept in the refrigerator. The stock solution was further diluted with methanol to obtain working solutions in the ranges of 65–800, 35–800, and 80–800 ng/mL for FXM, FLX, and PXT, respectively.

#### 3.4.2. Dosage Form Sample Solutions

For dosage form sample preparation, 10 tablets or the contents of 10 capsules were weighed and ground to a fine powder. An accurately weighed amount of the powder equivalent to 20 mg of the active ingredient was transferred into a 20 mL calibrated flask and dissolved in ~10 mL of distilled water. The solution was mixed well and then made up to the mark with water. The solution was filtered, and the filtrate was diluted with methanol to achieve suitable concentrations for analysis. These concentrations were in the ranges of 65–800, 35–800, and 80–800 ng/mL for FXM, FLX, and PXT, respectively.

#### 3.4.3. Plasma Samples

Aliquot (0.5 mL) of drug-free plasma dispensed into test tube, 50 µL of FXM and PXT solutions (500 ng/mL), and 2 mL of acetonitrile were added. After vortexing for 3 min, the tube was centrifuged at 4500 rpm for 20 min. The mixture was rendered alkaline with 0.1 M NaOH, and the alkaline solution was extracted and treated as described in a previous study [18]. Briefly, the samples were extracted three times with 3 × 1.5 mL dichloromethans:n-butanol (4:1, *v*/*v*) for FXM and FLX, and with hexane:isoamyl alcohol (99:1, *v*/*v*) for PXT. The extract was transferred to another 5 mL tube and evaporated to dryness under a stream of nitrogen gas at 40 °C. The residue was reconstituted, the samples were diluted, and the diluted samples were used for analysis using the proposed MW-SFA.

### 3.5. General Procedure of MW-SFA

Aliquots (150 μL) of SSRI solution of concentrations in the range of 35–500, 65–800, and 80–800 ng/mL for FLX, FXM, and PXT, respectively, were transferred into separate wells of the 96-well assay plates. Then, 50 μL of Clark and Lubs buffer solution of pH 8 for all SSRIs was added after 50 μL of NBD-Cl solution (0.2%, *w*/*v*). The plate was transferred to the microplate reader whose temperature control was adjusted to 50 ± 2 °C. The reactions were allowed to proceed for 20 min, and then 50 μL of HCl solution (0.1 M) was added to each well. The relative fluorescence intensity (RFI) of the resulting solution was measured at λ_ex_ = 470 nm, λ_em_ = 535 nm (for FXM), and at λ_ex_ = 490 nm, λ_em_ = 545 nm (for FLX and PXT). The blank wells were treated in a similar way, except 100 μL of water was dispensed in each well instead of the standard SSRI solution.

## 4. Conclusions

The present study describes the establishment of an MW-SFA for three SSRIs, namely, FLX, FXM, and PXT. The proposed assay involves the condensation reactions of the SSRIs, via their amino groups, with NBD-Cl reagent, resulting in the formation of fluorescent reaction products. The assay procedure was conducted in 96-well white opaque assay plates and the signals were measured using a microwell plate reader in fluorescence mode. Compared to all previously reported spectrofluorimetric assays for SSRIs, the proposed assay offers several advantages, including the use of small size samples/solvents, which is cost effective and environmentally friendly. Additionally, the assay is user-friendly, offers high throughput, and is suitable for the pharmaceutical and clinical analysis of SSRIs in dosage forms and plasma. The proposed assay is beneficial for the regular analysis of SSRIs in dosage forms, and it provides acceptable accuracy and precision. Moreover, it expands the utilization of microwell assays assisted with microplate readers for measuring various pharmaceuticals in the field of pharmaceutical and biomedical analysis.

## Figures and Tables

**Table 1:** Summary of the establishment of optimum conditions for the reaction of NBD-Cl with the investigated SSRIs.

Variable	Studied Range	Optimum Condition		
		FXM	FLX	PXT
NBD-Cl (%, *w*/*v*)	0.01–0.5	0.2	0.2	0.2
pH	5–9.5	8 ± 0.2	8 ± 0.2	8 ± 0.2
Temperature (°C)	25–50	50	50	50
Time (min)	5–60	20	20	20
HCl (M)	0.02–0.5	0.1	0.1	0.1
Solvent	Different ^a^	Methanol	Methanol	Methanol
Stability of SSRI-NBD (min)	10–60	60	60	60

^a^ Solvents tested: water, methanol, ethanol, isopropanol, acetone, and acetonitrile.

**Table 2:** Statistical parameters for the determination of SSRIs by the proposed MW-SFA based on their reaction with NBD-Cl reagent.

Parameter	FXM	FLX	PXT
λ_ex_ (nm)	470	490	490
λ_em_ (nm)	535	545	545
Linear range (ng/mL)	65–800	35–500	80–800
Intercept	4.3034	3.1802	6.3652
SD of intercept	1.5029	1.9249	1.7646
Slope	0.2366	0.5793	0.2304
SD of slope	0.0028	0.0084	0.0037
Correlation coefficient (r)	0.9995	0.9992	0.9993
LOD (ng/mL)	21	11	25
LOQ (ng/mL)	64	33	77

**Table 3:** Recovery studies for determination of SSRIs using the proposed MW-SFA based on their reaction with NBD-Cl.

Added SSRI (ng/mL)	Recovery (% ± SD) ^a^		
	FXM	FLX	PXT
100	98.5 ± 2.54	97.4 ± 2.09	100.2 ± 1.12
150	97.8 ± 2.02	98.1 ± 1.85	97.8 ± 1.85
200	100.1 ± 1.24	102.4 ± 2.24	99.4 ± 1.28
250	99.5 ± 2.55	99.6 ± 1.48	98.4 ± 1.06
300	101.4 ± 1.08	100.2 ± 1.88	101.2 ± 1.34

^a^ Values are mean of three determinations.

**Table 4:** Replicate analysis of SSRIs using the proposed MW-SFA based on their reaction with NBD-Cl reagent.

Sample Number	RFI ^a^		
	FXM (500 ng/mL)	FLX (200 ng/mL)	PXT (500 ng/mL)
1	112	121	118
2	108	115	113
3	110	117	108
4	105	110	115
5	110	120	116
Mean	109	116.6	114
SD	2.37	3.93	3.41
RSD (%)	2.17	3.37	2.99

^a^ RFI is the relative fluorescence intensity.

**Table 5:** Ruggedness of the proposed MW-SFA based on their reaction with NBD-Cl.

Parameters	Recovery (% ± RSD) ^a^		
	FXM	FLX	PXT
Instrument to instrument ^b^			
Instrument 1	96.3 ± 1.92	96.5 ± 1.82	100.5 ± 3.05
Instrument 2	98.4 ± 3.24	101.2 ± 2.72	101.2 ± 3.08
Day to day			
Day 1	95.8 ± 3.21	95.8 ± 3.70	99.2 ± 3.34
Day 2	101.4 ± 2.55	96.5 ± 2.96	95.5 ± 2.87
Day 3	97.2 ± 2.92	100.2 ± 1.88	102.4 ± 2.98

^a^ Values are the mean of three determinations ± SD.; ^b^ Instrument 1 was a SpectraMax^®^ M5 microplate reader (Molecular Devices, LLC, San Jose, CA, USA) and instrument 2 was an FLx800 fluorescence microplate reader (BioTek Instruments Inc., Winooski, VT, USA).

**Table 6:** Determination of SSRIs in their pharmaceutical dosage forms using the proposed MW-SFA and official assays.

Dosage Form ^a^	Label Claim (% ± SD) ^b^		F Value ^c^	t Value ^c^
	Proposed Assay	Official Assay ^d^		
Prozac capsules	100.2 ± 1.61	99.6 ± 1.22	2.63	1.74
Fluzac capsules	100.1 ± 1.61	99.7 ± 1.43	1.64	1.27
Salipax capsules	99.4 ± 1.54	99.8 ± 0.98	1.49	2.47
Flutin capsules	100.1 ± 1.32	100.1 ± 1.25	0.19	1.12
Octozac capsules	100.8 ± 1.83	101.2 ± 1.24	1.60	2.18
Faverin tablets	99.2 ± 1.45	99.5 ± 0.85	1.58	2.91
Seroxate tablets	99.7 ± 1.61	100.2 ± 0.93	2.38	3.00

^a^ The strengths of the dosage forms were given in the Experimental Section. ^b^ Values are mean of five determinations ± SD. ^c^ Theoretical values for t and F at 95% confidence limit (*n* = 5) were 2.78 and 6.39, respectively. ^d^ Reference [28] for FXM and reference [29] for FLX and PXT.

**Table 7:** Analysis of FXM and PXT in spiked plasma samples by the proposed MW-SFA.

Spiked Concentration (ng/mL)	FXM		PXT	
	Measured Conc. (ng/mL)	Recovery (% ± RSD) ^a^	Measured Conc. (ng/mL)	Recovery (% ± RSD) ^a^
80	78.5	98.1± 2.55	77.9	97.4 ± 2.41
120	121.7	101.4 ± 1.54	117.0	97.5 ± 1.85
240	241.2	100.5 ± 1.69	245.2	102.2 ± 2.97
500	489.0	97.8 ± 2.05	490.5	98.1 ± 1.56

^a^ Values are mean of three determinations.

## Data Availability

All data are available in the article.
